# Interface Charge Transfer Engineering in NiFe Layered Double Hydroxide-Cs_0.32_WO_3_ Heterostructures for Enhanced Oxygen Evolution Reaction

**DOI:** 10.3390/nano15161255

**Published:** 2025-08-14

**Authors:** Ze Wang, Xinyu Song, Yue Liu, Zhiwang Sun, Xin Zhang, Yuanhao Wang, Shifeng Wang

**Affiliations:** 1 Key Laboratory of Plateau Oxygen and Living Environment of Xizang Autonomous Region, College of Science, Xizang University, Lhasa 850000, China; wangze@stu.utibet.edu.cn (Z.W.); songxinyu@stu.utibet.edu.cn (X.S.); liuyue@stu.utibet.edu.cn (Y.L.); szh@stu.utibet.edu.cn (Z.S.); zhangxin@stu.utibet.edu.cn (X.Z.); 2 Hoffmann Institute of Advanced Materials, Shenzhen Polytechnic University, Shenzhen 518000, China

**Keywords:** oxygen evolution reaction, layered double hydroxides, heterostructure, interface charge transfer, electrocatalysis

## Abstract

Electrochemical water splitting for hydrogen production is considered a key pathway for achieving sustainable energy conversion. However, the sluggish reaction kinetics of the oxygen evolution reaction (OER) and high overpotentials severely hinder the large-scale application of water electrolysis technology. Nickel–iron layered double hydroxide (NiFe-LDH) has gained attention as a promising non-precious metal OER catalyst due to its abundant active sites and good intrinsic activity. However, its relatively low conductivity and charge transfer efficiency limit the improvement of catalytic performance. Therefore, this study used a simple hydrothermal method to generate a NiFe-LDH/Cs_0.32_WO_3_ heterojunction composite catalyst, relying on the excellent electronic conductivity of Cs_0.32_WO_3_ to improve overall charge transfer efficiency. According to electrochemical testing results, the modified NiFe-LDH/Cs_0.32_WO_3_-20 mg achieved a low overpotential of 349 mV at a current density of 10 mA cm^−2^, a Tafel slope of 67.0 mV dec^−1^, and a charge transfer resistance of 65.1 Ω, which represent decreases of 39 mV, 23.1%, and 40%, respectively, compared to pure NiFe-LDH. The key to performance improvement lies in the tightly bonded heterojunction interface between Cs_0.32_WO_3_ and NiFe-LDH. X-ray photoelectron spectroscopy (XPS) shows a distinct interfacial charge transfer phenomenon, with a notable negative shift of the W4f peak (0.85 eV), indicating the directional transfer of electrons from Cs_0.32_WO_3_ to NiFe-LDH. Under the influence of the built-in electric field within the heterojunction, this interfacial charge redistribution improved the electronic structure of NiFe-LDH, increased the proportion of high-valent metal ions, and significantly enhanced the OER reaction kinetics. This study provides new insights for the preparation of efficient heterojunction electrocatalysts.

## 1. Introduction

With the continuous growth of global energy demand and the increasingly severe environmental issues, seeking clean and sustainable energy conversion technologies has become a major challenge facing humanity [1,2]. Hydrogen energy, as an ideal clean energy carrier, has advantages such as high energy density and non-polluting combustion products, and is therefore widely regarded as a key technological pathway to achieving carbon neutrality [3,4]. Electrochemical water splitting technology can directly produce high-purity hydrogen using renewable electricity, thus providing the possibility for the large-scale development of the hydrogen industry [5,6,7]. However, the water electrolysis process includes two half-reactions: the hydrogen evolution reaction (HER) and the oxygen evolution reaction (OER) [8,9,10]. OER involves a complex four-electron transfer process and multiple reaction intermediates (*OH, *O, *OOH), with slow reaction kinetics and requiring high overpotentials, severely affecting the overall water electrolysis efficiency [11,12,13]. Therefore, developing high-activity, low-overpotential OER electrocatalysts is crucial for promoting the commercialization of water electrolysis technology.

Currently, commercial electrolyzers typically use precious metal-based IrO_2_ or RuO_2_ as catalysts for the OER process [14,15,16]. Although they exhibit excellent electrocatalytic activity, their high cost and difficulty in mass production greatly limit their large-scale use [17]. Therefore, the research of non-precious metal OER catalysts based on abundant elements on Earth has become a hot topic. Among many materials, NiFe-LDH belongs to a class of typical layered double hydroxides with a unique layered anionic clay structure, which can be represented by the formula [M^2+^_1-x_M^3+^_x_(OH)_2_]^x+^(A^n−^)_x/n_·mH_2_O, where M^2+^ and M^3+^ represent divalent and trivalent metal ions, respectively. In NiFe-LDH, Ni^2+^ ions mainly provide a large number of active sites, while Fe^3+^ ions effectively modulate the electronic structure of the Ni sites, reducing the adsorption energy of intermediates and optimizing the OER reaction pathway [18,19]. More importantly, NiFe-LDH undergoes surface reconstruction in alkaline electrolyte to form the γ-NiOOH and FeOOH active phases, which are considered the true OER active centers [20]. However, the inherent low conductivity and limited charge transfer efficiency of NiFe-LDH limit its catalytic performance at high current densities [21,22]. To solve this problem, many strategies have been proposed, including element doping [23], defect engineering [24], and the construction of heterostructures [25]. Among them, the heterostructure strategy, by combining LDH with materials having excellent electronic conductivity, not only improves the overall charge transfer performance but also generates synergistic effects at the interface, developing additional active sites [26].

Although the strategy of constructing heterostructures holds significant potential for improving the performance of LDH-based catalysts, it still faces many challenges. On one hand, how to select the second component to better combine with NiFe-LDH lacks specific reference standards. Traditional composite methods often focus solely on the improvement of a single property, such as conductivity or the number of active sites, while neglecting the impact of interface effects on overall performance [27]. On the other hand, the charge transfer mechanism at the heterojunction interface is still unclear, especially how the reorganization of the interface electronic structure affects the intrinsic activity of active sites and the selectivity of reaction pathways [28]. Tungsten bronze materials (MₓWO_3_, where M is an alkali metal or alkaline earth metal) are a class of non-stoichiometric oxides with unique electronic properties. Cs_0.32_WO_3_ is a type of tungsten bronze material with a hexagonal tungsten bronze structure, where Cs^+^ ions occupy the hexagonal channels formed by WO_6_ octahedra. This structure enables Cs_0.32_WO_3_ to exhibit metallic conductivity, with a conductivity at room temperature reaching 10^3^–10^4^ S cm^−1^, which is much higher than that of most metal oxides. Moreover, the coexistence of multiple oxidation states of W atoms (W^4+^, W^5+^, W^6+^) gives it good electronic transport capability and chemical stability [29]. However, despite the wide application of tungsten bronze materials in fields such as electrochromism and energy storage, research on combining them with LDH materials to form heterojunctions for electrocatalysis is scarce. Therefore, exploring the composite mechanism of NiFe-LDH and tungsten bronze materials and elucidating how interfacial charge transfer affects electrocatalytic performance is of great scientific significance and practical value for designing efficient heterojunction OER catalysts.

Based on the above challenges, this study successfully synthesized a NiFe-LDH/Cs_0.32_WO_3_ heterojunction composite catalyst through a simple hydrothermal method and investigated its OER electrocatalytic performance and mechanism. The Cs_0.32_WO_3_ tungsten bronze was introduced into the NiFe-LDH composite system to fully utilize the excellent electronic conductivity of Cs_0.32_WO_3_ to improve the catalyst’s charge transfer efficiency. The electronic structure was regulated through the heterointerface to promote the generation of high-valent active species. A directional electron transfer model from Cs_0.32_WO_3_ to NiFe-LDH was established. This provides a theoretical foundation for the interfacial effects of heterojunction catalysts. The results show that the NiFe-LDH/Cs_0.32_WO_3_-20 mg composite catalyst exhibits excellent OER performance, with an overpotential of only 349 mV (10 mA cm^−2^), a decrease of 39 mV compared to pure NiFe-LDH, and a Tafel slope reduced to 67.0 mV dec^−1^. The charge transfer resistance also decreased to 65.1 Ω. XPS analysis showed significant charge redistribution at the interface. The large negative shift of the W4f peak (0.85 eV) indicates the electron transfer process from Cs_0.32_WO_3_ to NiFe-LDH. This interfacial charge transfer effectively improves the electronic structure of the active sites and increases the proportion of high-valent metal ions, while the high-valent metal ions serve as the true active sites for OER by facilitating the formation of key intermediates and lowering the energy barrier for O_2_ evolution, thereby significantly enhancing the electrocatalytic activity of NiFe-LDH. This study not only provides an effective strategy for developing efficient non-precious metal OER catalysts but also contributes important scientific insights into the understanding of the heterojunction interfacial charge transfer mechanism.

## 2. Materials and Methods

### 2.1. Synthesis of NiFe-Layered Double Hydroxides and Their Derivatives

#### 2.1.1. Materials

All the chemical reagents used in this study were of analytical grade and used directly without further purification. Nickel nitrate hexahydrate (Ni(NO_3_)_2_·6H_2_O, analytical grade) was purchased from Tianjin Damo Chemical Reagents Factory (Tianjin, China). Ferric nitrate nonahydrate (Fe(NO_3_)_3_·9H_2_O, purity 99.99%), ammonium fluoride (NH_4_F, analytical grade, purity 96%), urea (CO(NH_2_)_2_, analytical grade, purity 99%), potassium hydroxide (KOH, purity 95%), tungsten hexachloride (WCl_6_, purity 99%), cesium hydroxide monohydrate (CsOH·H_2_O, analytical grade), glacial acetic acid (CH_3_COOH, analytical grade), and Nafion D-521 dispersion (5% by weight) were all purchased from Shanghai Aladdin Biochemical Technology Co., Ltd. (Shanghai, China). Anhydrous ethanol (C_2_H_5_OH, analytical grade) was provided by Chengdu Jinshan Chemical Reagents Co., Ltd. (Chengdu, China). Deionized water (resistivity 18.25 MΩ cm^−1^) was prepared using the ULUPURE (UPR-II-10TNZ) system and used throughout all experiments.

#### 2.1.2. Preparation of Cs_0.32_WO_3_

Cs_0.32_WO_3_ material was prepared by the solvothermal method. First, 0.18 g of cesium hydroxide monohydrate (CsOH·H_2_O) was added to 32 mL of anhydrous ethanol, followed by the addition of 1.28 g of tungsten hexachloride (WCl_6_), and stirred until completely dissolved. Then, 8 mL of glacial acetic acid was slowly added as a reducing agent, and the mixture was stirred to generate a uniform precursor solution. The solution was transferred into a 100 mL Teflon-lined high-pressure reactor and reacted at a constant temperature of 230 °C for 24 h. After the reaction system naturally cooled, the blue-black solid product was collected by centrifugation, and residual organic solvents and unreacted substances were removed. Finally, the washed product was dried in a 60 °C oven for 12 h to obtain the Cs_0.32_WO_3_ material.

#### 2.1.3. Preparation of NiFe-LDH

NiFe-LDH material was synthesized via a one-step hydrothermal method. First, 1.8 mmol of Ni(NO_3_)_2_·6H_2_O, 0.9 mmol of Fe(NO_3_)_3_·9H_2_O, 64 mmol of CO(NH_2_)_2_, and 13.5 mmol of NH_4_F were dissolved in 50 mL of deionized water, and the mixture was magnetically stirred at room temperature for 2 h to form a uniform solution. The solution was then placed into a 100 mL stainless steel high-pressure reactor lined with Teflon, and reacted at a constant temperature of 105 °C for 12 h. After the reaction system naturally cooled, the mixture was centrifuged and washed several times to remove unreacted materials. The resulting precipitate was dried under vacuum at 60 °C for 12 h to obtain the NiFe-LDH material.

#### 2.1.4. Preparation of NiFe-LDH/Cs_0.32_WO_3_

NiFe-LDH/Cs_0.32_WO_3_ composite materials were synthesized using a simple hydrothermal method. Based on the previous NiFe-LDH synthesis system, different amounts of Cs_0.32_WO_3_ powder (10 mg, 20 mg, 30 mg, 40 mg, and 50 mg) were added to the mixed solution containing metal salt precursors and additives. The mixture was thoroughly stirred to ensure uniform dispersion of Cs_0.32_WO_3_ in the reaction system. The reaction was then carried out at 105 °C for 12 h, followed by standard washing and drying treatments. A series of composite materials with different Cs_0.32_WO_3_ loading amounts were obtained. These are NiFe-LDH/Cs_0.32_WO_3_-10 mg, NiFe-LDH/Cs_0.32_WO_3_-20 mg, NiFe-LDH/Cs_0.32_WO_3_-30 mg, NiFe-LDH/Cs_0.32_WO_3_-40 mg, and NiFe-LDH/Cs_0.32_WO_3_-50 mg.

### 2.2. Characterizations and Testing

#### 2.2.1. Characterizations

The crystalline phase structure was characterized using a Bruker D8 Advance powder (Bruker Corporation, Billerica, MA, USA) diffractometer with Cu Kα radiation (wavelength 1.54059 Å). The microstructure of the samples was observed using a CIQTEK SEM5000 electron microscope (CIQTEK Co., Ltd., Hefei, China). Elemental distribution and chemical composition were analyzed by mapping using a Thermo Fisher Apreo C field emission scanning electron microscope (Thermo Fisher Scientific Inc., Waltham, MA, USA) coupled with energy dispersive spectroscopy. High-resolution transmission electron microscope images were obtained using an FEI Tecnai G2 F30 (FEI Company, Hillsboro, OR, USA). Surface chemical states and valence state information were obtained using a Thermo Fisher Escalab 250XI X-ray photoelectron spectrometer (Thermo Fisher Scientific Inc., Waltham, MA, USA). The Al Kα monochromator (photon energy 1486.6 eV) was used with the following testing parameters: X-ray power 150 W, analysis area 650 µm, accelerating voltage 14.8 kV, and emission current 1.6 A. The energy spectrum calibration was referenced to the binding energy of the contaminant carbon C1s at 284.8 eV.

#### 2.2.2. Electrocatalytic Performance Testing

The OER performance was tested using a standard three-electrode system on a CHI660E electrochemical workstation (CH Instruments, Inc., Austin, TX, USA). The electrolyte was a 1 M KOH solution. The electrode system included a 3 mm diameter glassy carbon working electrode (geometric area S = πr^2^ = 0.0706 cm^2^), a Hg/HgO reference electrode, and a graphite rod counter electrode. All electrochemical data were converted to potentials relative to the reversible hydrogen electrode (RHE), using the conversion formula E_RHE_ = E_Hg/HgO_ + 0.923 V. The testing procedure was as follows: first, 20 cycles of cyclic voltammetry were performed at a scan rate of 100 mV/s for pretreatment to ensure electrode activation and stabilization. This pretreatment process removes surface contaminants, promotes the formation of active species, and allows the electrode to reach a stable electrochemical state. Then, linear sweep voltammetry was performed in the range of 0–0.8 V vs. RHE at a scan rate of 5 mV/s to obtain the polarization curve. Finally, impedance testing was performed at the potential corresponding to a current density of 10 mA/cm^2^, with a frequency range of 100 kHz to 1 Hz.

## 3. Results

**Figure 1:**
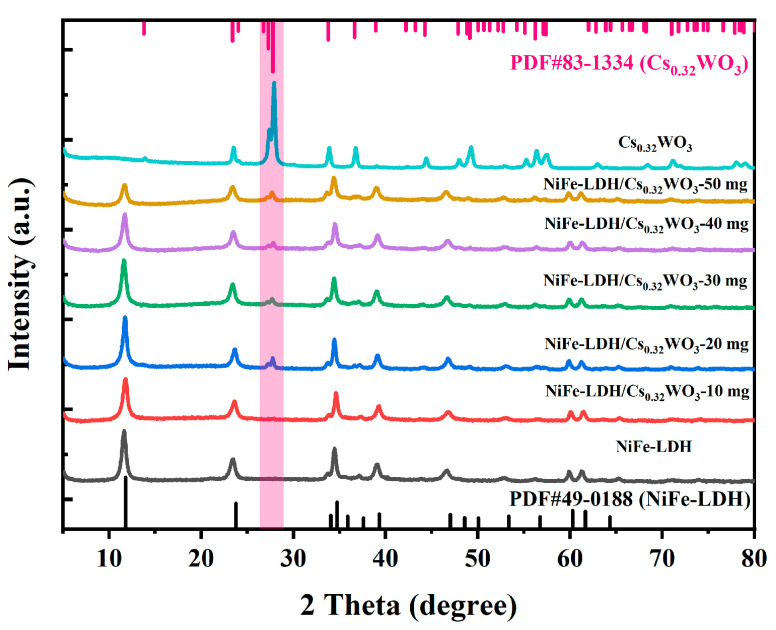
XRD patterns of NiFe-LDH, Cs_0.32_WO_3_, and NiFe-LDH/Cs_0.32_WO_3_ composites with different Cs_0.32_WO_3_ loadings (10–50 mg).

Figure 1 shows the XRD patterns of Cs_0.32_WO_3_, NiFe-LDH, and NiFe-LDH/Cs_0.32_WO_3_ composite materials. From the diffraction pattern of NiFe-LDH, it can be seen that NiFe-LDH was successfully synthesized, and it matches well with the standard card PDF#49-0188, exhibiting a hexagonal crystal system with a layered structure (a = b = 3.066 Å, c = 22.471 Å, α = β = 90°, γ = 120°) [30]. The characteristic diffraction peaks appear at 11.6°, 23.5°, 34.5°, 39.1°, and 46.7°, corresponding to the (003), (006), (012), (015), and (018) crystal planes, respectively. The diffraction pattern of Cs_0.32_WO_3_ matches the standard card PDF#83-1334, also exhibiting a hexagonal crystal system (a = b = 7.4116 Å, c = 7.5981 Å, α = β =90°, γ = 120°). The major characteristic peaks at 23.5°, 27.4°, 27.9°, 36.7°, and 49.3° correspond to the (002), (102), (200), (202), and (220) crystal planes, respectively, proving the successful preparation of the cesium tungsten bronze phase. In the diffraction patterns of NiFe-LDH/Cs_0.32_WO_3_ composite materials, as the loading amount of Cs_0.32_WO_3_ increased from 10 mg to 50 mg, all composite samples retained the diffraction peak characteristics of NiFe-LDH. This indicates that the intrinsic layered structure of NiFe-LDH was not destroyed during the hydrothermal composite process. On the other hand, the intensity of the main peak of NiFe-LDH at 11.6° (003) gradually decreases with the increasing loading of Cs_0.32_WO_3_. More importantly, when the loading amount of Cs_0.32_WO_3_ exceeds 20 mg, significant diffraction peaks appear at 27.3° and 27.8° in the NiFe-LDH/Cs_0.32_WO_3_ composite material, which correspond to the (102) and (200) crystal planes of Cs_0.32_WO_3_, confirming the successful composite of the two materials.

**Figure 2:**
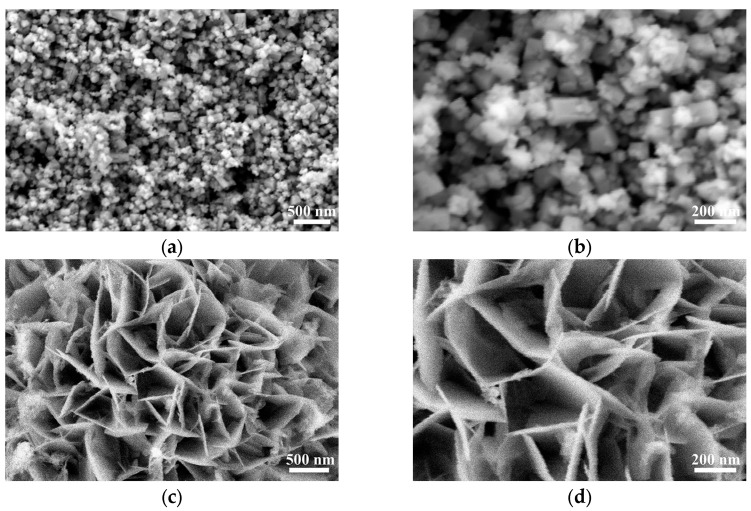

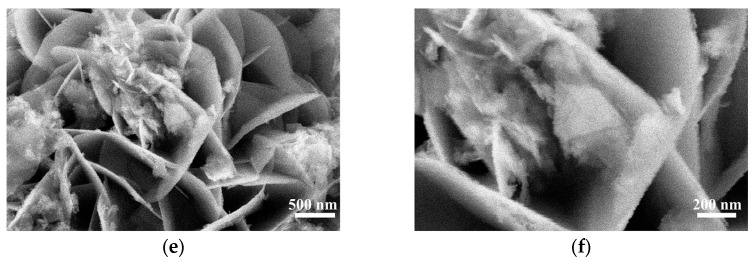
SEM images of different samples at various magnifications: (**a**,**b**) Cs_0.32_WO_3_, (**c**,**d**) NiFe-LDH, and (**e**,**f**) NiFe-LDH/Cs_0.32_WO_3_-20 mg composite.

Figure 2 shows the SEM images of different samples at different magnifications, which reveal the microstructural features of the materials. As shown in Figure 2a,b, Cs_0.32_WO_3_ exhibits a nanoparticle aggregation morphology, with particle sizes approximately 200 nm, and the particle surfaces are relatively smooth with clear boundaries. The morphology of pure NiFe-LDH, as shown in Figure 2c,d, presents the typical nanosheet structure of layered double hydroxides. These nanosheets exhibit high anisotropy and form a flower-like structure composed of nanosheets. This unique layered porous structure not only provides a large number of surface active sites but also creates conditions for the rapid diffusion of electrolyte and timely desorption of gas products. In the NiFe-LDH/Cs_0.32_WO_3_-20 mg composite material (Figure 2e,f), we can clearly observe the composite of the two materials. The composite material still retains the basic nanosheet structural framework of NiFe-LDH, with dispersed Cs_0.32_WO_3_ nanoparticles observed on the surface of the nanosheets and in the interlayer spaces. Moreover, the Cs_0.32_WO_3_ particles are relatively dispersedly distributed on the NiFe-LDH nanosheets, and no obvious aggregation phenomenon is observed. This also indicates good interfacial compatibility between Cs_0.32_WO_3_ and NiFe-LDH during the hydrothermal composite process. After the composite, the material maintains the original porous structure characteristics, and due to the space-filling effect between the two phases, a more intricate hierarchical porous structure is formed. This unique composite morphology not only retains many of the catalytic active sites of NiFe-LDH, but also introduces the electronic conductivity of Cs_0.32_WO_3_, which plays a key role in building an efficient electron transport network and achieving synergistic electrocatalytic effects. Moreover, the heterogeneous interface between the two phases generates new interfacial active sites, thereby improving the electrocatalytic performance of the composite material. These new interfacial active sites arise from the combination of different components that can induce electronic redistribution and achieve synergistic effects at the interface, potentially generating additional active centers at these contact regions [31].

**Figure 3:**
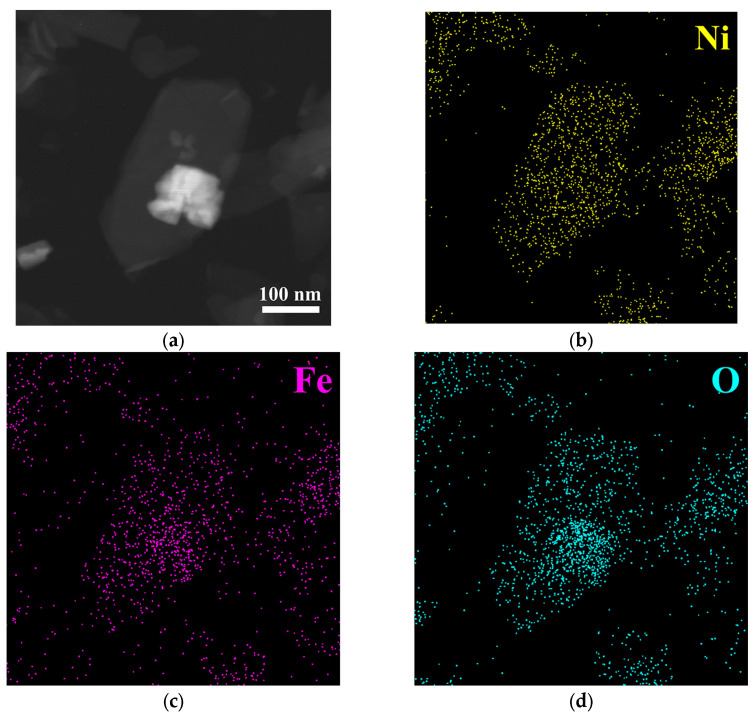

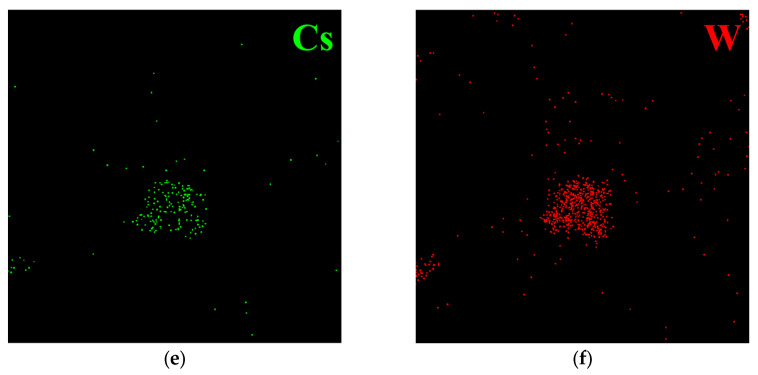
Elemental mapping of (**a**) NiFe-LDH/Cs_0.32_WO_3_-20 mg and the distribution of (**b**) Ni, (**c**) Fe, (**d**) O, (**e**) Cs, and (**f**) W.

The EDS element distribution map in Figure 3 shows the distribution of various elements in the composite material, with Ni and Fe elements mainly distributed in the nanosheet region, fully corresponding to the spatial distribution of NiFe-LDH, while Cs and W elements are distributed in the particle region, confirming the presence of the Cs_0_._32_WO_3_ phase. The O element is distributed across the entire region because both phases contain oxygen elements. The spatial correspondence of the element distribution confirms the successful composite of NiFe-LDH and Cs_0_._32_WO_3_. To further determine the precise composition of the composite material, a quantitative TEM-EDS analysis was performed on NiFe-LDH/Cs_0.32_WO_3_-20 mg (Figure S1), and the elemental composition was as follows: O (70.2%), Ni (12.6%), Fe (10.0%), W (5.4%), Cs (1.8%). The W and Cs content (7.2 wt%) indicates that the Cs_0.32_WO_3_ component has been successfully doped into the composite structure.

**Figure 4:**
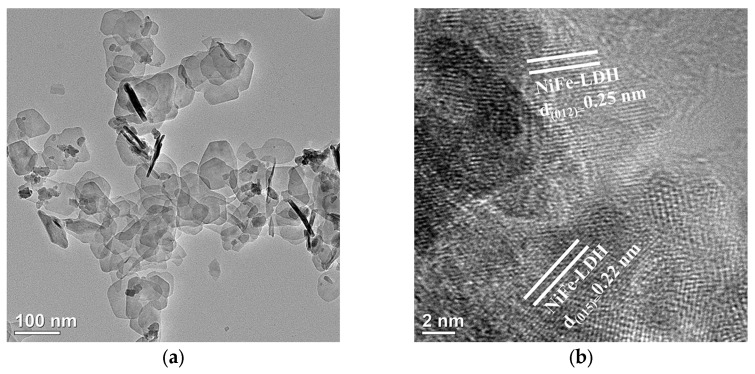

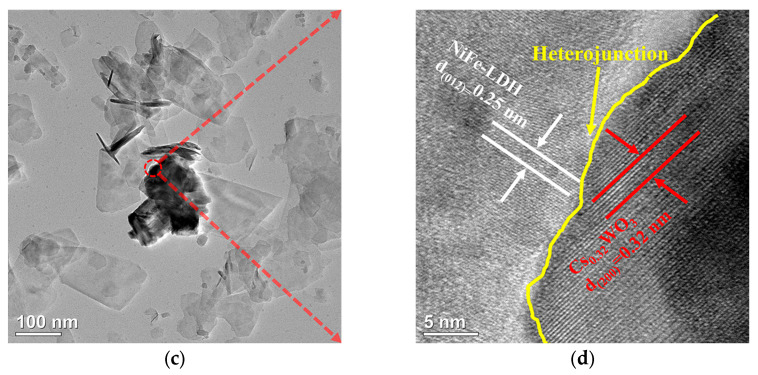
(**a**,**b**) TEM and HRTEM images of NiFe-LDH, (**c**,**d**) TEM and HRTEM images of NiFe-LDH/Cs_0.32_WO_3_-20 mg composite.

The TEM and HRTEM analyses in Figure 4 further reveal the microstructure of the materials and the interface composite situation. Figure 4a shows the TEM morphology of pure NiFe-LDH, where the ultra-thin nanosheet structure is clearly visible, matching the intrinsic structural features of layered double hydroxides. The HRTEM image in Figure 4b provides evidence of the lattice structure of NiFe-LDH, where the observed interplanar spacings of 0.22 nm and 0.25 nm correspond to the (015) and (012) crystal planes of NiFe-LDH, respectively. This indicates the successful preparation of the nanosheet structure of NiFe-LDH. In the NiFe-LDH/Cs_0_._32_WO_3_-20 mg composite material (Figure 4c), the TEM image further confirms the successful composite of the two phases. In the composite material, the basic nanosheet morphology of NiFe-LDH is observed, and some dark granular material is visible on the surface and edges of the nanosheets, which belongs to the Cs_0_._32_WO_3_ component. The HRTEM image in Figure 4d shows lattice fringes of both materials, with the 0.25 nm fringe corresponding to the (012) crystal plane of NiFe-LDH, and the 0.32 nm lattice fringe corresponding to the (200) characteristic crystal plane of Cs_0_._32_WO_3_. There is also a distinct heterojunction interface between the two phases. This tight heterojunction interface facilitates fast charge carrier transfer and creates additional interfacial active sites, which has a positive effect on enhancing the electrocatalytic performance.

**Figure 5:**
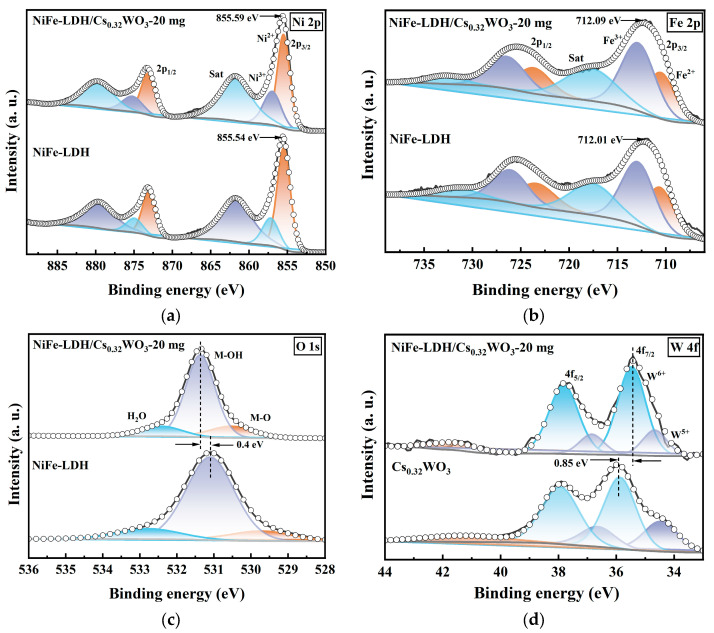
XPS spectra of NiFe-LDH and NiFe-LDH/Cs_0.32_WO_3_-20 mg composite: (**a**) Ni 2p, (**b**) Fe 2p, (**c**) O 1s, and (**d**) W 4f spectra.

XPS analysis (Figure 5) further investigates the changes in the electronic structure and the interfacial charge transfer during the composite process of NiFe-LDH and Cs_0.32_WO_3_. In the Ni2p spectrum (Figure 5a), the Ni2p_3/2_ peak of the composite material shifts positively from 855.54 eV to 855.59 eV relative to NiFe-LDH, indicating that Ni atoms lose some electrons and adjust toward a higher oxidation state. In the Fe2p spectrum (Figure 5b), the Fe2p_3/2_ peak also shifts slightly towards a higher binding energy relative to NiFe-LDH, indicating that Fe atoms are also involved in the electron transfer process, while the proportion of high-valent Ni and Fe species increases. The O1s spectrum of pure NiFe-LDH (Figure 5c) can be fitted into three components: the peak at 529.7 eV corresponds to metal–oxygen bonds (M–O), the peak at 531.1 eV corresponds to metal–hydroxyl bonds (M–OH), and the broad peak at 532.8 eV is attributed to surface-adsorbed water molecules (H_2_O). The O1s peak of the composite material shifts 0.4 eV positively compared to NiFe-LDH, indicating that the chemical environment of the oxygen atoms has changed and the electron density has decreased. A significant change is observed in the W4f spectrum (Figure 5d), where the W4f_7/2_ peak of the composite material shifts negatively by 0.85 eV compared to pure Cs_0.32_WO_3_. This obvious chemical shift indicates that electrons transfer from Cs_0.32_WO_3_ to NiFe-LDH, with W atoms, as electron donors, losing electrons, resulting in a decrease in binding energy, while Ni, Fe, and O atoms, as electron acceptors, gain electrons. This interfacial charge redistribution forms a built-in electric field at the interface between the two phases, thereby promoting the rapid migration of charge carriers and lowering the OER reaction energy barrier. The increase in high-valent metal ions also provides more electrocatalytic active sites, and therefore, the electron transfer from Cs_0.32_WO_3_ to NiFe-LDH not only confirms the strong electronic interaction between the two phases but also provides the necessary electronic structural basis for the improvement of the composite material’s electrocatalytic performance.

**Figure 6:**
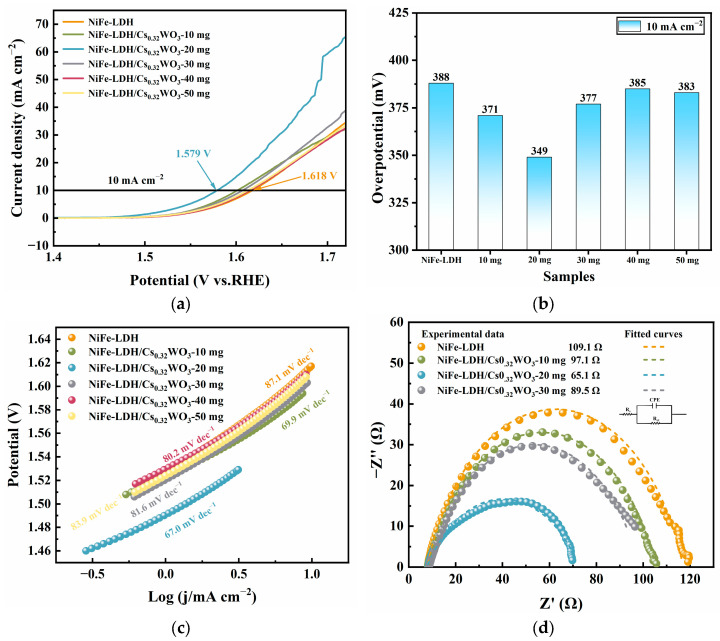
Electrochemical performance of NiFe-LDH and NiFe-LDH/Cs_0.32_WO_3_ composites with different Cs_0.32_WO_3_ loadings for OER: (**a**) LSV polarization curves, (**b**) overpotential comparison at 10 mA cm^−2^, (**c**) Tafel slopes, and (**d**) EIS Nyquist plots with equivalent circuit model.

Figure 6 evaluates the OER electrochemical performance of NiFe-LDH and composite materials with different Cs_0.32_WO_3_ loading amounts. The linear sweep voltammetry (LSV) polarization curves (Figure 6a) show differences in the electrocatalytic activity of the samples. Pure NiFe-LDH exhibits an overpotential of 388 mV at a current density of 10 mA cm^−2^. After adding Cs_0.32_WO_3_, the composite materials with different doping amounts show improvements compared to NiFe-LDH. When the Cs_0.32_WO_3_ loading reaches 20 mg, the composite material performs the best, with the polarization curve shifting noticeably to the left, generating a higher current density at the same potential. To better understand the role of each component in the composite material, we also tested the OER performance of pure Cs_0.32_WO_3_ (Figure S2). As shown in the figure, the OER activity of pure Cs_0.32_WO_3_ is poor, much lower than that of NiFe-LDH and the composite material. This indicates that Cs_0.32_WO_3_ itself is not an effective OER catalyst; its main role in the composite material is to enhance the catalytic activity of NiFe-LDH through interfacial charge transfer effects. The overpotential comparison (Figure 6b) also shows this improvement, with the overpotential of NiFe-LDH/Cs_0.32_WO_3_-20 mg being 349 mV, 39 mV lower than pure NiFe-LDH. However, when the Cs_0.32_WO_3_ loading increases further to 30–50 mg, the overpotential increases instead. This suggests that there is an optimal composite ratio, and an excessive amount of Cs_0.32_WO_3_ may cover the active sites of NiFe-LDH. Tafel slope analysis (Figure 6c) provides key information about the reaction kinetics mechanism. The Tafel slope of pure NiFe-LDH is 87.1 mV dec^−1^, while NiFe-LDH/Cs_0.32_WO_3_-20 mg shows the lowest Tafel slope (67.0 mV dec^−1^), indicating that it has the fastest kinetics. A smaller Tafel slope is often associated with more efficient charge transfer and better intermediate adsorption energy. Electrochemical impedance spectroscopy (EIS) analysis (Figure 6d) reveals the intrinsic mechanism of the charge transfer process. The Nyquist plots of all samples exhibit typical semicircular features, with the diameter reflecting the charge transfer resistance (Rct). From the equivalent circuit fitting results, it can be seen that the charge transfer resistance of pure NiFe-LDH is 109.1 Ω. After adding Cs_0.32_WO_3_, the charge transfer resistance of the composite materials significantly decreases, and NiFe-LDH/Cs_0.32_WO_3_-20 mg has the smallest Rct, only 65.1 Ω, which is 40% lower than that of pure NiFe-LDH. This significant decrease in resistance directly demonstrates the positive effect of Cs_0.32_WO_3_’s excellent electronic conductivity on the overall charge transfer efficiency. Figure S3 shows a comparison of the cyclic voltammograms of NiFe-LDH and NiFe-LDH/Cs_0.32_WO_3_-20 mg composite material, both of which exhibit distinct oxidation peaks around 1.40 V vs. RHE, corresponding to the oxidation process of Ni^2+^ to Ni^3+^. However, the oxidation peak current of the composite material increases, and the peak potential shifts slightly negatively, indicating that the addition of Cs_0.32_WO_3_ is beneficial for the formation of high-valent active species [32]. Durability and stability are another key factor in evaluating the performance of catalyst materials. As shown in Figure S4, the prepared NiFe-LDH/Cs_0.32_WO_3_-20 mg composite catalyst demonstrates excellent long-term electrochemical stability, operating stably for 50 h at a current density of 10 mA cm^−2^ in a 1 M KOH electrolyte.

To better evaluate the performance of our composite catalyst, Table S1 presents a performance comparison between NiFe-LDH/Cs_0.32_WO_3_-20 mg and other reported non-precious metal OER electrocatalysts [33,34,35,36]. The results indicate that the NiFe-LDH/Cs_0.32_WO_3_-20 mg electrocatalyst we prepared outperforms other currently reported non-precious metal catalysts in OER performance, showing great potential for practical applications.

## 4. Conclusions

In this study, NiFe-LDH/Cs_0.32_WO_3_ heterojunction composite catalysts were successfully synthesized via a hydrothermal method, and the mechanism for the enhancement of OER electrocatalytic performance was explained. Structural characterization showed that Cs_0.32_WO_3_ nanoparticles were uniformly dispersed on the NiFe-LDH nanosheets, forming a tight heterojunction interface. XPS analysis revealed the directional electron transfer process from Cs_0.32_WO_3_ to NiFe-LDH. The notable negative shift of the W4f peak (0.85 eV) and the positive shifts of the Ni, Fe, and O peaks collectively indicated interfacial charge redistribution. This charge transfer formed a built-in electric field at the heterojunction interface, optimizing the electronic structure of active sites and increasing the proportion of high-valent metal species. Electrochemical tests showed that NiFe-LDH/Cs_0.32_WO_3_-20 mg exhibited the best OER performance, with the overpotential decreasing from 388 mV of pure NiFe-LDH to 349 mV (a decrease of 39 mV). The Tafel slope was 67.0 mV dec^−1^ (a reduction of 69%), and the charge transfer resistance decreased by 40%, mainly due to the excellent electronic conductivity of Cs_0.32_WO_3_, which formed an efficient charge transfer network. This facilitated interfacial charge transfer, reduced the activation energy barrier for interfacial charge transfer, and increased the active sites at the heterojunction interface. This study provides deep insights into the interfacial charge transfer mechanism in heterojunction catalytic materials and offers an effective approach for further development of high-performance water electrolysis electrocatalysts.

## Supplementary Materials

The following supporting information can be downloaded at: https://www.mdpi.com/article/10.3390/nano15161255/s1, Figure S1: Quantitative TEM-EDS spectrum of NiFe-LDH/Cs_0.32_WO_3_-20mg composite material. Figure S2: LSV polarization curve of pure Cs_0.32_WO_3_ for OER in 1 M KOH electrolyte. Figure S3: Cyclic voltammograms of NiFe-LDH and NiFe-LDH/Cs_0.32_WO_3_-20mg. Figure S4: Chronopotentiometric curve of NiFe-LDH/Cs_0.32_WO_3_-20 mg at current density of 10 mA cm^−2^. Table S1: Comparison table of different reported non-noble metal electrocatalysts with NiFe-LDH/Cs_0.32_WO_3_-20mg.
