# Supplementary material for: Interface Charge Transfer Engineering in NiFe Layered Double Hydroxide-Cs0.32WO3 Heterostructures for Enhanced Oxygen Evolution Reaction
Source: Nanomaterials (Basel). 2025 Aug 14;15(16):1255. doi: 10.3390/nano15161255 (PMC12388688; doi:10.3390/nano15161255)
Supplement: Supplementary file 1 [file nanomaterials-15-01255-s001.zip › nanomaterials-3787818-supplementary.pdf]

Interface Charge Transfer Engineering in NiFe Layered Double Hydroxide-$Cs_{0.32}WO_3$ Heterostructures for Enhanced Oxygen Evolution Reaction

Ze Wang, Xinyu Song, Yue Liu, Zhiwang Sun, Xin Zhang, Yuanhao Wang and Shifeng Wang

Supporting Information

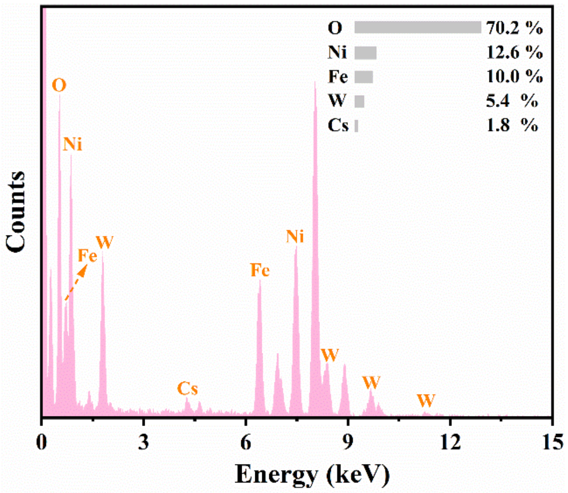

Figure S1. Quantitative TEM-EDS spectrum of NiFe-LDH/$Cs_{0.32}WO_3$-20mg composite material.

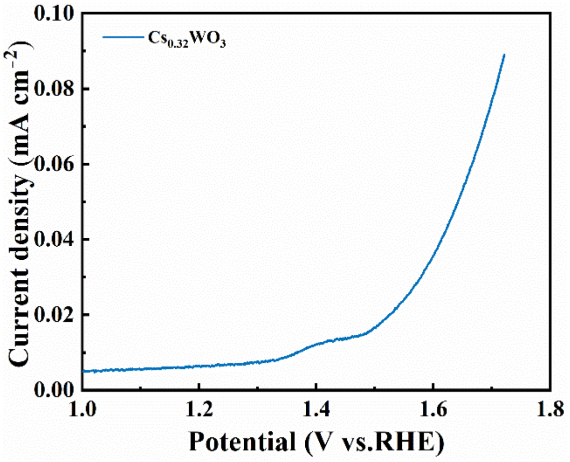

Figure S2. LSV polarization curve of pure $Cs_{0.32}WO_3$ for OER in 1 M KOH electrolyte.

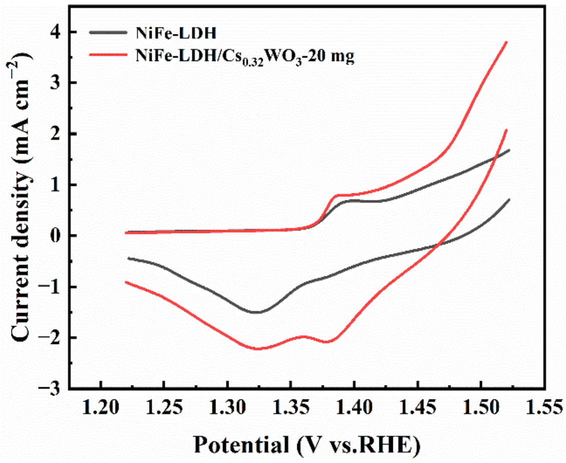

Figure S3. Cyclic voltammograms of NiFe-LDH and NiFe-LDH/$Cs_{0.32}WO_3$-20mg.

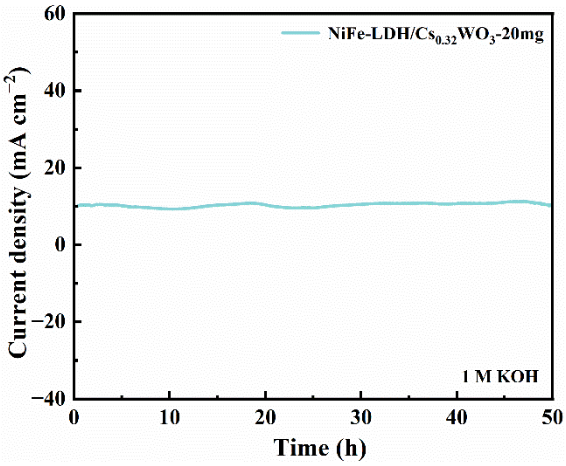

Figure S4. Chronopotentiometric curve of NiFe-LDH/$Cs_{0.32}WO_3$-20 mg at current density of 10 mA cm^{-2}.

Table S1. Comparison table of different reported non-noble metal electrocatalysts with NiFe-LDH/$Cs_{0.32}WO_3$-20mg.

Electrocatalysts	Overpotential at 10 mA cm^{-2}	Tafel Slope (mV dec^{-1})	Reference
NiFeC LDH	360 mV	72	[1]
RuO_2-CeO_2	350 mV	74	[2]
Ni-CAT/NiFe-LDH/ CNFs	370 mV	79	[3]
Co_2P NP	364 mV	78	[4]
NiFe-LDH/$Cs_{0.32}WO_3$-20mg	349 mV	67	This work

References

1. Berger, M.; Popa, I. M.; Negahdar, L.; Palkovits, S.; Kaufmann, B.; Pilaski, M.; Hoster, H.; Palkovits, R., Elucidating the Influence of Intercalated Anions in NiFe LDH on the Electrocatalytic Behavior of OER: A Kinetic Study. *ChemElectroChem* **2023**, 10, e202300235.
2. Galani, S. M.; Mondal, A.; Srivastava, D. N.; Panda, A. B., Development of RuO_2/CeO_2 heterostructure as an efficient OER electrocatalyst for alkaline water splitting. *Int. J. Hydrogen Energy* **2020**, 45, 18635-18644.

3. Li, J.; Qin, Y.; Bai, Z.; Li, S.; Li, L.; Ouyang, B.; Kan, E.; Zhang, W., Investigating the role of 3D hierarchical Ni-CAT/NiFe-LDH/CNFs in enhancing the oxygen evolution reaction and Zn-air battery performance. *Appl. Surf. Sci.* **2024**, 648, 159080.
4. Jebaslinhepzybai, B. T.; Partheeban, T.; Gavali, D. S.; Thapa, R.; Sasidharan, M., One-pot solvothermal synthesis of Co_2P nanoparticles: An efficient HER and OER electrocatalysts. *Int. J. Hydrogen Energy* **2021**, 46, 21924-21938.
